# Good publication practices in clinical pharmacology: transparency, evidence-based medicine and the 7-D assessment*

**DOI:** 10.5414/CP202421

**Published:** 2015-09-02

**Authors:** Barry G. Woodcock, Veronika Luger

**Affiliations:** Dustri Medical Science Publications, Munich-Oberhaching, Germany

**Keywords:** good publication practices, clinical pharmacology, evidence-based medicine, 7-D assessment

## Abstract

Transparency and evidence-based medicine are cornerstones of good publication practices (GPP), and concern publishers, editors, research investigators, and reviewers alike. Methods for implementing these principles within the framework of GPP are described. The main aspects include obtaining a *Manuscript Agreement Contract*, a *Statement on Transparency of Authorship* and a *Declaration of Conflicts of Interest* from the authors. Assessing whether a manuscript meets the requirements of EBM is demonstrated using the “7-D assessment”. The main purpose of this tool is to established that the (1) right **D**esign, (2) right **D**iagnosis, (3) right **D**rug molecule, (4) right **D**osage, (5) right **D**ata, (6) right **D**eductions, and (7) right **D**ocumentation have been implemented in order to meet the objectives of the investigation. If the findings from any one of these assessments is questionable, the compliance of the research with EBM principles will be weakened and the reviewers and editors will make recommendations to the publisher accordingly. The guidelines described will help to provide a fair and transparent process of scientific publication and foster the freedom of clinical pharmacological research.

*This text is based on an internal report outlining how the principles of evidence-based medicine can be incorporated into good publication practices: Publication of research findings in medicine and the Editorial Policy of the International Journal of Clinical Pharmacology and Therapeutics by B.G. Woodcock and V. Luger. Dustri Medical Science Publications. Dustri-Verlag Dr. Karl Feistle GmbH & Co. KG, Munich-Oberhaching, Germany and Dustri-Verlag Inc., Rockledge, FL, USA, www.dustri.com, July 2015. 

## Introduction 

Good publication practices (GPP) concern publishers, editors, research investigators, and reviewers alike. This is because the publication of research findings in the medical sciences is not driven by altruism or an innate human need to alleviate suffering. Authors of publications in medical research on the one hand, are almost always motivated by the desire for recognition and/or financial factors, publishers and editors no less so. Thus, those involved directly in the publishing process, especially authors, are required to exercise absolute integrity. Indeed, clinical pharmacology manuscripts frequently contain recommendations for drug treatment as well as other information having a direct bearing on patient health and well-being. Since publishers and editors carry responsibility for the material they publish, the application of GPP and the contribution made by peer reviewers are of considerable importance

1. to ensure complete transparency of authorship and
2. to ensure that the principles of evidence-based medicine (EBM) have been applied.

This viewpoint focuses on the implementation of these GPP (see also COPE, 2008 [1]). 

## A) Good publication practices and transparency 

Editors, referees, and the publisher in particular are interested in the questions:

Who funded the work?
Who did the work?
Has the work been published before?
Has the study been registered?
Have ethical issues been addressed correctly?
Has the peer review system been applied in an appropriate manner?
Are there issues of plagiarism or copyright?
Are there ethnic factors to consider?

Ignoring these questions could have a deleterious effect on a journal’s image and impact factor and therefore a *Manuscript Agreement Contract*, a *Statement on Transparency of Authorship,* and a *Declaration of Conflicts of Interest* will be required. 

### 1) Manuscript Agreement Contract

A *Manuscript Agreement Contract* in which answers to most of these questions are given will need to be made between authors and publisher. It incorporates a statement from the authors confirming

- that the investigation described in the manuscript is not being considered for publication elsewhere – no double-submission,
- that all authors named agree to publication of the manuscript,
- that publication copyright passes to the publisher.

(Further reading: See Research Methods and Reporting in Graf C et al. 2009 [2] and Bareket-Samish et al. 2009 [3]). 

### 2) Statement on Transparency of Authorship

A statement confirming transparency of authorship describes the contribution of each author to the work being submitted. The list of authors should include all those who have contributed directly to the research. 

A statement from authors certifying that their manuscript has not been sponsored by a commercial entity and the absence of any acknowledgements means that non-authors have made no substantial contribution to the manuscript. 

### 3) Declaration of Conflicts of Interest

A conflict of interests is a set of circumstances that creates a risk that professional judgment or actions regarding a primary interest will be unduly influenced by a secondary interest. 

Authors submitting manuscripts to a journal for publication are required to disclose all of the authors’ relationships with companies that make products studied or discussed in the article, companies that make related products, and other pertinent entities with an interest in the topic. If no relationships exist with companies that make products studied or discussed in the article then this can be indicated explicitly. 

In summary,

- if the authors receive payment or other type of reward for any work done,
- if a commercial enterprise makes a payment, donates materials, equipment, services or drugs to a hospital, commercial research unit or academic institution or finances the statistical evaluation of data or makes payments to patients this must be declared by giving full details of the nature of the sponsorship and name and address of the sponsor(s).

What is sometimes forgotten is that the same holds true for reviewers. It is essential that they inform the publisher of any possible conflict of interest as outlined above. In addition, they should inform the publisher of any personal relationship to the authors that could influence the review. If a conflict of interest exists, the reviewer must decline to provide a review. 

In other cases, the existence of a conflict of interest sends out the message that the findings in the published work must be interpreted with caution for the reasons given above. 

## B) Good publication practices and evidence-based medicine 

Sackett et al. 1966 [4] defined EBM as *“….the conscientious, explicit, and judicious use of current best evidence in making decisions about the care of individual patients. The practice of evidence based medicine means integrating individual clinical expertise with the best available external clinical evidence from systematic research*.” Clinical pharmacology manuscripts should therefore include a patient-driven search and appraisal of the available literature and incorporate the best available evidence as EBM in the manner described by Sackett et al. [3]. Most authors of research articles in clinical pharmacology are aware of this and use facts derived from basic research, individual clinical expertise and the best available external evidence when presenting their findings. 

The publisher delegates to editors and they in turn to peer reviewers the task of assessing the scientific quality of submitted research. A main aspect of this task must be to ensure as far as possible that the principles of evidence-based medicine have been applied. 

The peer review of manuscripts is carried out by editors, members of the editorial board and referees with experience in the relevant fields who are invited to peer review for the journal. Names of suitable referees can be suggested by authors. As a general rule at least two reviewers’ reports are obtained before a decision on publication is reached. A final decision on acceptance is usually made by the Editor-in-Chief, a Section Editor, or by an International Editor within a time-frame that respects the interests of the author. 

Assessing whether a manuscript meets the requirements of EBM need not be difficult if the “7-D assessment” method, put forward here as a suggestion, is used and this holds true for most studies demonstrating drug efficacy, so called “negative studies” where a drug is shown to have no effect on a chosen clinical parameter, e.g., in QT studies, bioavailabilty and pharmacokinetic studies, reviews and case reports. 

The “7-D Assessment” comprises:

1. right **D**esign
2. right **D**iagnosis,
3. right **D**rug molecule,
4. right **D**osage,
5. right **D**ata,
6. right **D**eductions
7. right **D**ocumentation

where

1. *right design* means that the study design and protocol are appropriate for answering the question(s) being asked;
2. *right diagnosis* is relevant for investigations both in patients and healthy subjects where subject and patient description and patient selection need to be detailed, accurate and appropriate for the aims of the study;
3. *right drug molecule* begs the questions “Is the active agent a known molecular species?” and “Can the drug entity have a mode of action compatible with the observed pharmacological effects? Has a pharmacological effect observed in vitro a counterpart in vivo? Do confounding factors exist such as the presence of drug enantiomers, stereoisomers or drug combinations? Herbal drugs and extracts do not generally fit in with the concepts of EBM. High first pass effects make it likely that more than one active species is present in the tissues;
4. *right dosage* concerns not only the size of the dose (i.e., Is the dose or concentration clinically relevant?), but also the method of administration, bioavailability, and duration of treatment. These questions also apply to in-vitro studies with tissues and cells;
5. *right data* are those data required to meet the objectives of the study, which can establish or disprove efficacy, which have been obtained using state-of-the-art methods, and which have been evaluated using recognized data analysis procedures. In the case of reviews of the literature, the retrieval methods used and quality of the studies reviewed would need to be scrutinized;
6. *right deductions* means that conclusions will be made following a correct and objective interpretation of the research findings and should address safety and efficacy requirements in clinical pharmacotherapy;
7. *right documentation* addresses primarily the quality of the evidence in the supportive literature and asks the questions “Is the documentation up-to-date? Is it obtained from peer-reviewed sources, and is it comprehensive?” The citation of websites is very useful for providing information, but must be viewed with caution when used to provide evidence. Information on websites is not peer-reviewed and can be subject to change.

If the finding of any one of these assessments is questionable, the compliance of the research with EBM principles will be weakened and the reviewers and editors will make recommendations to the publisher accordingly. 

Ignoring these questions might have a deleterious effect on the integrity and credibility of the publication process the importance of which was referred to in the first paragraph above. 

## Conclusion 

Transparency and EBM are cornerstones of GPP and methods for implementing these principles within the framework of GPP have been described. They will help to provide a fair and transparent process of scientific publication and foster the freedom of clinical pharmacological research. 

## Conflicts of interest 

Barry G. Woodcock is Editor-in-Chief of the International Journal of Clinical Pharmacology and Therapeutics. Veronika Luger is an Editorial Officer employed by Dustri-Verlag Dr. Karl Feistle GmbH & Co. KG, Munich-Oberhaching, Germany. 
